# Analysis of Clinical Outcome and Predictors of Mortality in Pediatric Trauma Population: Evidence from a 10 Year Analysis in a Single Center

**DOI:** 10.3390/children8080688

**Published:** 2021-08-10

**Authors:** Ya-Chih Yang, Tsung-Han Hsieh, Chi-Yuan Liu, Chun-Yu Chang, Yueh-Tseng Hou, Po-Chen Lin, Yu-Long Chen, Da-Sen Chien, Giou-Teng Yiang, Meng-Yu Wu

**Affiliations:** 1 Department of Emergency Medicine, Taipei Tzu Chi Hospital, Buddhist Tzu Chi Medical Foundation, New Taipei 231, Taiwan; foxcat721@yahoo.com.tw (Y.-C.Y.); brianann75@gmail.com (Y.-T.H.); taipeitzuchier@gmail.com (P.-C.L.); yulong0129@gmail.com (Y.-L.C.); sam.jan1978@msa.hinet.net (D.-S.C.); gtyiang@gmail.com (G.-T.Y.); 2 Department of Emergency Medicine, School of Medicine, Tzu Chi University, Hualien 970, Taiwan; 3 Department of Research, Taipei Tzu Chi Hospital, Buddhist Tzu Chi Medical Foundation, New Taipei 231, Taiwan; tch28047@tzuchi.com.tw; 4 Department of Orthopedic Surgery, Taipei Tzu Chi Hospital, Buddhist Tzu Chi Medical Foundation, New Taipei 231, Taiwan; cy.liu@tzuchi.com.tw; 5 Department of Orthopedics, School of Medicine, Tzu Chi University, Hualien 970, Taiwan; 6 Department of Anesthesiology, Taipei Tzu Chi Hospital, Buddhist Tzu Chi Medical Foundation, New Taipei 231, Taiwan; paulchang1231@gmail.com; 7 School of Medicine, Tzu Chi University, Hualien 970, Taiwan

**Keywords:** shock index, pediatric age-adjusted shock index, trauma, pediatric trauma, mortality

## Abstract

The shock index (SI) is a useful tool for predicting the injury severity and mortality in patients with trauma. However, pediatric physiology differs from that of adults. In the pediatric trauma population, the shock status may be obscured within the normal range of vital signs. Pediatric age-adjusted SI (SIPA) is reported more accurately compared to SI. In our study, we conducted a 10 year retrospective cohort study of pediatric trauma population to evaluate the SI and SIPA in predicting mortality, intensive care unit (ICU) admission, and the need for surgery. This retrospective cohort study included 1265 pediatric trauma patients from January 2009 to June 2019 at the Taipei Tzu Chi Hospital, who had a history of hospitalization. The primary outcome of this investigation was in-hospital mortality, and the secondary outcomes were the length of hospital and ICU stay, operation times, and ICU admission times. The SIPA group can detect changes in vital signs early to reflect shock progression. In the elevated SIPA group, more severe traumatic injuries were identified, including high injury severity score (ISS), revised trauma score (RTS), and new injury severity score (NISS) scores than SI > 0.9. The odds ratio of elevated SIPA and SI (>0.9) to predict ISS ≥ 16 was 3.593 (95% Confidence interval [CI]: 2.175–5.935, *p* < 0.001) and 2.329 (95% CI: 1.454–3.730, *p* < 0.001). SI and SIPA are useful for identifying the compensatory phase of shock in prehospital and hospital settings, especially in corresponding normal to low-normal blood pressure. SIPA is effective in predicting the mortality and severity of traumatic injuries in the pediatric population. However, SI and SIPA were not significant predictors of ICU admission and the need for surgery analysis.

## 1. Introduction

Despite the advances in medical care, it is observed that ten individuals die due to trauma injuries every minute [1]. Recently, deaths due to trauma have increased and pose a considerable financial burden on health insurance. Based on the current concept of the American College of Surgeons Committee on Trauma (ACS-COT), early accurate prediction of traumatic injury severity could be transferred to higher-level facilities and provide total care for every aspect of the injury. The shock index (SI, the ratio of heart rate to systolic blood pressure) has been reported as a sensitive marker of hemodynamic instability to reflect the shock severity. In previous adult studies, an SI > 0.9 is shown to predict transfusion needs and mortality [2,3]. However, few studies have focused on pediatric populations. The direct application of adult trauma scores to the pediatric population is not suitable [4,5]. In the study by N. Acker et al., [6] the adjusting of SI is promoted by adjusting the age-based pediatric vital signs to provide a higher accuracy than unadjusted SI. This pediatric age-adjusted shock index (SIPA) has been validated in a few studies [6,7]. Other studies have evaluated the role of SI and SIPA in predicting intensive care unit (ICU) admissions. Compared to other symptom scores, SIPA could be calculated by the emergency department or emergency medical services (EMS) without pediatric weight or advanced intervention parameters. In prehospital evaluations, SIPA provided an easy access for emergency medical technicians to predict the traumatic injury severity. However, there is a lack of strong evidence for SIPA in the pediatric population with trauma. Therefore, in our study, we conducted a 10 year retrospective cohort study of pediatric population with trauma to evaluate SI and SIPA in predicting mortality, ICU admission, and operation (OP).

## 2. Methods

### 2.1. Study Design and Inclusion Criteria

This was a retrospective cohort study using the Taipei Tzu Chi Hospital trauma database from January 2009 to June 2019 and was approved by the Institutional Review Board of Taipei Tzu Chi Hospital (IRB number: 10-XD-072). The Taipei Tzu Chi Hospital trauma database contains 152 data elements related to trauma patients and hospital information, including detailed patient demographics, prehospital medical conditions, vital signs, in-hospital vital signs, abbreviated injury scale (AIS) score, injury severity score (ISS), and in-hospital and in-emergency department (ED) mortality. We included all pediatric trauma patients aged ≤20 years from January 2009 to June 2019 who visited Taipei Tzu Chi Hospital and had a history of hospitalization. The primary outcome of this investigation was in-hospital mortality, and the secondary outcomes were the length of hospital and ICU stay, OP times, and ICU admission times. The exclusion criteria included (1) patients who were diagnosed with an out-of-hospital cardiac arrest, (2) patients whose clinical outcome or important data were missing, and (3) patients who had no hospitalization. Several trauma scoring systems have been reported to evaluate the trauma severity and predict clinical outcomes. However, there is less evidence to support these findings in the pediatric population. Therefore, we investigated the current trauma score systems in the pediatric population, including the ISS, Glasgow coma scale (GCS), revised trauma score (RTS), SI, National Industrial Security System (NISS), and new trauma and injury severity score (TRISS). A subgroup analysis was also conducted to analyze the traumatic score systems in different age and injury types.

### 2.2. Shock Index and Pediatric Age-Adjusted Shock Index

The SI, a physiological triage score, was calculated using the recorded heart rate (HR) and systolic blood pressure (SBP) using the following formula: SI = HR/SBP. The SI is a sensitive marker for predicting the shock status, which has been studied in multiple populations, including sepsis, cardiovascular disease, and obstetric population [8]. The normal range for the SI is reported as 0.5–0.7. Some evidence suggests that an SI higher than 0.9 is acceptable to believe hemodynamic instability in patients. Pediatric physiology differs from adults, and the normal range of pediatric vital signs varies with age, which could significantly influence the SI values. Therefore, the SIPA has been proposed to identify and predict hemodynamically unstable children [6]. The cut-off values and normal ranges of SIPA are listed in Table 1.

### 2.3. Statistical Analysis

The demographic details, overall survival, and clinical outcome data were analyzed using the SPSS software (Version 13.0 SPSS Inc, Chicago, IL, USA) for statistical analysis. All continuous variables are reported as the mean, standard deviation (SD), and median. Categorical variables were reported as numbers with percentages. Continuous variables were compared using the independent sample t-test for normally distributed data and the Mann–Whitney U test for non-normally distributed data. The categorical variables were compared using the Pearson chi-squared test or Fisher’s exact test. Multivariable logistic regression was used to analyze the clinical outcomes in pediatric trauma patients. The variables with *p* < 0.10, or important variables, were selected for multivariable logistic regression analysis. The area under the receiver operating characteristic curve (AUROC) was used for each outcome to analyze the discrimination of the regression model. All tests were two-sided, and a *p* value < 0.05 was considered as statistically significant.

## 3. Results

**Figure 1:**
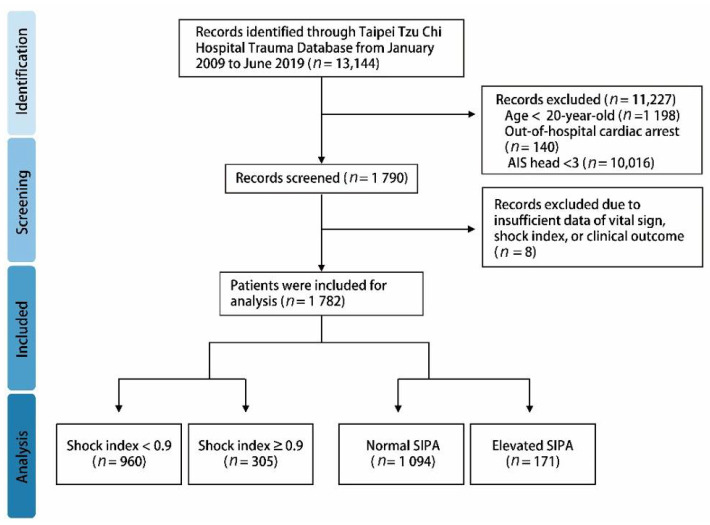
Schematic diagram illustrating the detailed inclusion of pediatric trauma patients.

A total of 1265 patients were identified in the Taipei Tzu Chi Hospital trauma database from January 2009 to June 2019. A detailed flowchart is shown in Figure 1. In the included pediatric trauma population (Table 2), the mean age was 14 years, and 72.1% of the population were boys. The triage distribution of patients was observed to be 55.2% in level II, followed by level III (36.9%). The consciousness level in the emergency department was 91.1% with total alertness (GCS: 15) and severe coma status in 24 patients (1.9%). Of a total of 1265 patients, 760 patients (60.1%) were injured in the street, 268 patients (21.2%) were injured in a public site, and 160 patients (12.6%) were injured at home. The major injury mechanism was traffic accidents (53.1%), followed by the secondary injury mechanism of pediatric trauma. The extremities were the major injury sites, accounting for up to 72.7% of injuries, followed by the head (18.8%) and facial (15.7%) injuries. Fifty-eight pediatric patients were activated by the trauma team. In traumatic score systems, 305 patients presented a high SI, and 171 patients showed an elevated SIPA. Seventy-eight patients had an ISS ≥ 16. In the clinical outcome analysis, the median hospital length of stay (LOS) was 5 days, and 177 patients were admitted to the ICU. Surgical intervention was needed in 867 patients, and 49 patients underwent reoperation. In total, seven patients died during the in-hospital follow-up (Table 3). In the population with a high SI level and elevated SIPA, the blood pressure was significantly lower with respect to the systolic and diastolic blood pressure, associated with higher respiratory and heart rates. These findings reflected an early unstable hemodynamic status. The severe injury population, such as ISS ≥ 16 and high ISS and RTS score, is significant in the abnormal SI and elevated SIPA group. In addition, the high SI level group showed a longer LOS, and the elevated SIPA group had a high mortality rate (Table 4).

**Figure 2:**
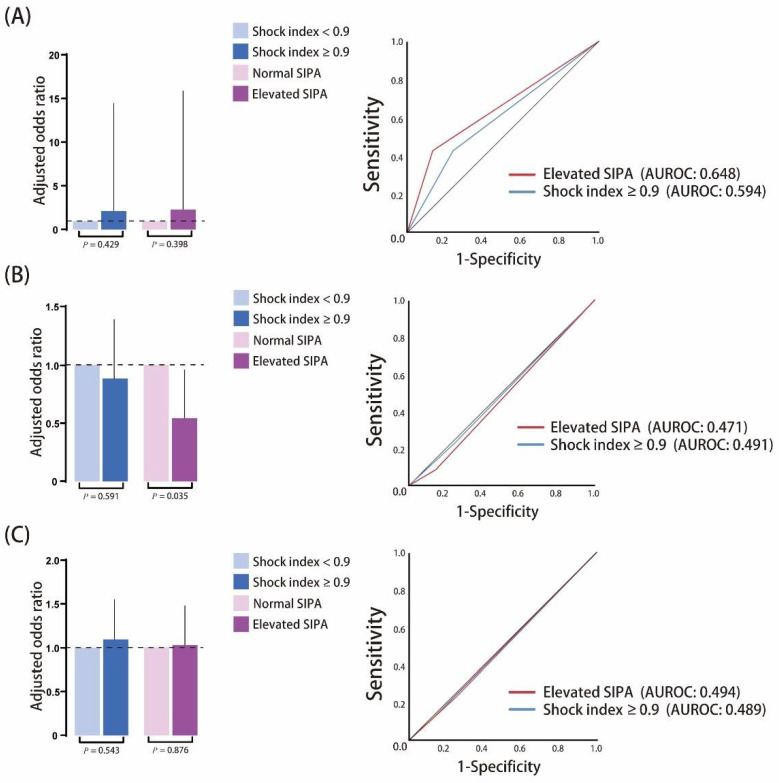
Schematic diagram illustrating the adjusted odds ratio of elevated shock index (SI) > 0.9 and pediatric age-adjusted SI (SIPA) group in (**A**) mortality, (**B**) intensive care unit (ICU) admission, and (**C**) need for surgery with area under the receiver operating characteristic curve (AUROC).

**Figure 3:**
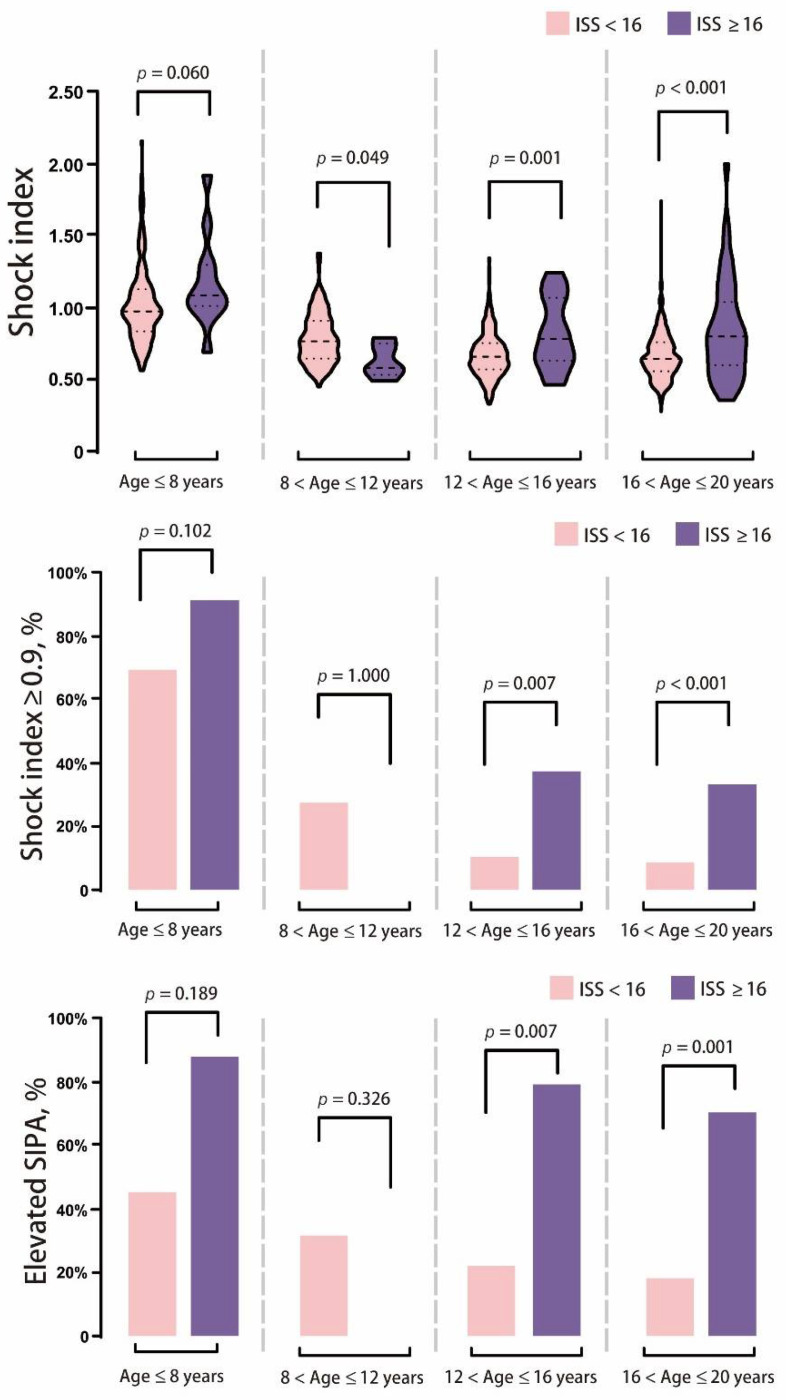
Schematic diagram illustrating the shock index (SI), the percentage of SI ≥ 0.9 and elevated pediatric age-adjusted SI (SIPA) of distribution of high (injury severity score, [ISS] ≥ 16) and low injury (ISS < 16) in different age range.

**Figure 4:**
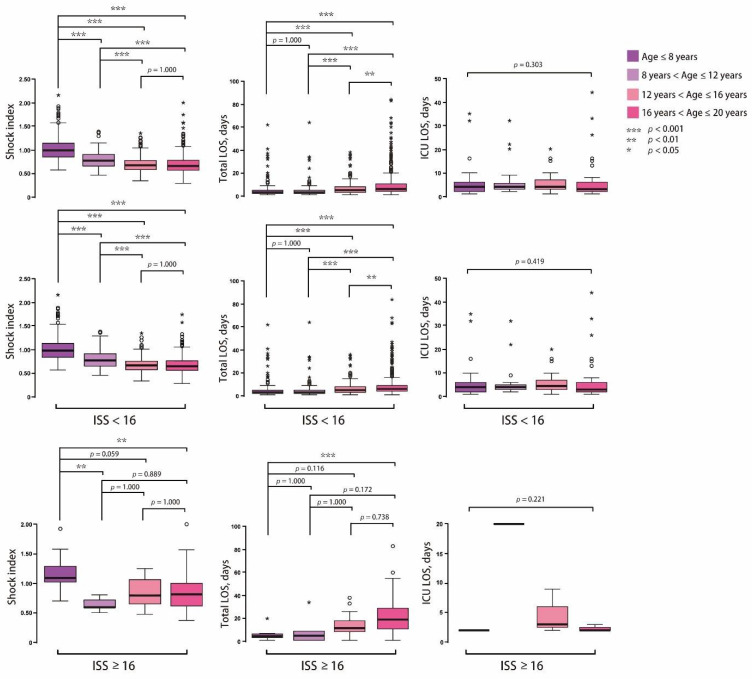
The distribution of shock index, total length of stay (LOS), and intensive care unit (ICU) LOS in different age in high (injury severity score, [ISS] ≥ 16) and low injury (ISS < 16).

**Figure 5:**
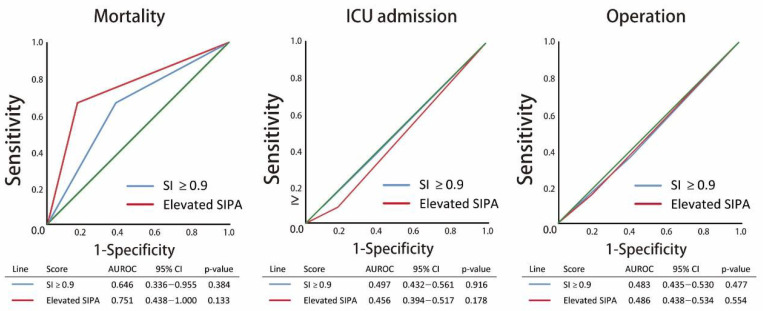
The area under the receiver operating characteristic curve (AUROC) of shock index > 0.9 and elevated pediatric age-adjusted SI (SIPA) predicting mortality, intensive care unit (ICU) admission, and need for surgery in population with age ≤ 16.

All the quantitative variables considered to have potential correlations with the mortality, ICU admission, and the need for surgery showed statistically significant associations, including age, past history, diastolic blood pressure, and the severity of injury (elevated SIPA, ISS, RTS, NISS, and TRISS). To compare the SI value > 0.9 with elevated SIPA, we performed a quantitative assessment of associations by performing an odds ratio (OR) analysis by adjusting for age, past history, diastolic pressure, and severity of injury (ISS ≥ 16). For mortality, the adjusted OR of SI > 0.9 was 2.151 (95% Confidence interval [CI]: 0.322–14.362, *p*-value: 0.429) and 2.295 in the elevated SIPA group (95% CI: 0.334–15.777, *p* = 0.398). The AUROC was 0.594 in the SI > 0.9 group and 0.648 in the elevated SIPA group. In the prediction of ICU admission, the adjusted OR of SI > 0.9 showed 0.883 (95% CI: 0.560–1.392, *p* = 0.591) and 0.542 in the elevated SIPA group (95% CI: 0.306–0.958, *p* = 0.035). The AUROC was 0.491 in the SI > 0.9 group and 0.471 in the elevated SIPA group. In predicting the need for surgery, the adjusted OR of SI > 0.9 was 1.110 (95% CI: 0.794–1.551, *p* = 0.543) and 1.029 in the elevated SIPA group (95% CI: 0.719–1.473, *p* = 0.876). The AUROC was 0.489 in the SI > 0.9 group and 0.494 in the elevated SIPA group (Figure 2). In the subgroup analysis, home was the major injury site in the population aged ≤8 years. As the age increased, injury at the street increased from 27.1% to 82.3%. In our study, the patients aged between 8 and 12 years had a lower ISS and percentage of ISS ≥ 16 compared to other groups. The mean SI was higher in the age group ≤ 8 years, followed by the 8–12 years group. The young population has up to 70.8% patients with a SI ≥ 0.9; however, only 22.5% of the population was observed to have elevated SIPA. SI was observed to decrease with age, and the percentage of SI ≥ 0.9 is the same. Although the percentage of elevated SIPA also increased with age, the trend was slower than that of SI. In the outcome analysis, the total LOS and the percentage of deaths increased in the high age population (Table 5). In all the patients, the SI of ISS ≥ 16 was significantly higher than the ISS < 16 (mean ± SD: 0.88 ± 0.33 vs. 0.76 ± 0.23). Similar results were observed in the populations of ≤ 8 years, 12–16 years, and 16–20 years. The percentage of SI > 0.9, and elevated SIPA was also significantly higher in the patients with ISS ≥ 16 than in those with ISS < 16 (Figure 3). The total LOS was longer in the severe injury group with ISS ≥ 16; however, the ICU LOS was not significant (Figure 4). Based on the criteria of Acker et al., [6], the patients aged ≤16 years were included for analysis of SIPA and SI > 0.9, predicting the mortality, ICU admission, and the need for surgery. The AUROC curve of elevated SIPA was better than SI > 0.9 in patients aged ≤16 years (AUROC: 0.751 vs. 0.646) (Figure 5).

## 4. Discussion

An accuracy prediction tool for the shock status is important for physicians and emergency medical technicians to assess the severity of diseases. Several studies have investigated the predictive capability of SI in a population with trauma and compared it with the traditional vital signs, serum biomarkers, and other scoring systems [9]. In the trauma population, the SI has been investigated in hemorrhagic shock for the early recognition of the need for fluid resuscitation. Compared to normal vital signs, SI may be present in the early shock phase, such as the compensatory phase of shock. In the current concept, early resuscitation in the “golden hour” could correct the vicious cycle of hemorrhage injury to prevent an “early death”. Therefore, a novel marker to detect hemodynamic instability is effective for early intervention, including activation of the massive transfusion protocol, trauma team, transcatheter arterial embolization (TAE), and resuscitative endovascular balloon occlusion of the aorta (REBOA). In a prospective study by Birkhahn et al. [10], 46 healthy blood donors were included, and 450 mL of blood was removed for 20 min. Although the HR was elevated and SBP was lower, the change in the vital signs was still within the normal range. However, the mean SI was significantly higher. Another retrospective cohort study that analyzed 8111 blunt trauma patients showed that a higher SI was significantly associated with the need for massive transfusion (risk ratio: 8.13, 95% CI: 4.60–14.36) [11]. A similar result was reported by DeMuro et al. [12]. However, in pediatric trauma patients, the SI was not suitable for reflecting hemodynamic instability due to the different physiology of adults. The SIPA by an adjusted normal range of pediatric vital signs has been proposed to predict the outcomes and presented more accurately identified children with shock status. In the study by Acker et al. [6], 543 children with severe blunt injury were included, and the results showed that more severe pediatric trauma patients were identified via elevated SIPA than SI > 0.9, especially in need for transfusion, high-grade liver/spleen laceration, high ISS score, and a high in-hospital mortality rate. Our study showed similar results. The elevated SIPA group can detect changes in vital signs early to reflect shock progression, even if vital signs were within the normal range. An elevated SIPA can signify more severe traumatic injuries, including high ISS, RTS, and NISS scores than SI > 0.9. In our study, the odds ratio of elevated SIPA and SI > 0.9 to predict ISS ≥ 16 was 3.593 (95% CI: 2.175–5.935, *p* < 0.001) and 2.329 (95% CI: 1.454–3.730, *p* < 0.001). These findings suggest that SIPA is more specific than the vital signs or SI alone in predicting the severity of traumatic injury in a pediatric population. In the clinical outcome analysis, an elevated SIPA has a high in-hospital mortality rate. Although SIPA and SI are both effective in predicting the in-hospital mortality, there were no cases of ICU admission and the need for surgery. In previous studies, SIPA was reported to be a better predictor of ICU admission, in-hospital mortality, need for surgery, endotracheal intubation, and blood transfusion [6,7,13]. There are several reasons for this result. First, SIPA is an acute marker which reflected “early death” in the pediatric trauma population. The shock status may be corrected by adequate resuscitation. An initial elevated SIPA is a hint for physicians for an early intervention to prevent shock progression, which may impair the predictive capability of other outcomes in SIPA. In our study, several pediatric trauma patients received early fluid resuscitation to correct abnormal SIPA; additionally, serial follow-up SIPAs were within normal limits. They also did not require other interventions. We believe that a serial elevated SIPA, such as during the first 24 h of admission, is more reliable for predicting ICU admission, in-hospital mortality, and the need for surgery. Second, traumatic injury-induced shock may not only cause hemorrhage. The pediatric airway obstruction caused hypoxia, such as face injury, hemothorax or pneumothorax, may not reflect the shock sign by elevated SIPA. A traumatic brain injury is another important issue that may present with bradycardia, irregular respiration, and widened pulse pressures (Cushing’s triad) due to increased intracranial pressure. In our analysis, head and neck injuries accounted for up to 251 patients (18.8%). This may impair the sensitivity of the SIPA to predict the shock status. Finally, pediatric patients are usually irritable and anxious when vital signs are observed. The environmental stress and pain sensations from the traumatic injury stimulate the sympathetic nervous system, leading to tachycardia and hypertension. This may impair the accuracy of the SIPA in predicting the shock status.

This study had several limitations. First, there is an inevitable source of bias in the measurement of the vital signs and triage by different people. In our database, we did not repeat the measurements of vital signs or by the same staff. Second, every patient in the emergency department received different treatment orders, which may have impaired the clinical outcomes, even if the severity of traumatic injury was similar. Third, the serial SIPA or SI was not recorded. In our database, the in-hospital vital signs are only obtained in the emergency department. Therefore, we did not perform SIPA and SI analyses after 24 h or at admission. Finally, this retrospective study did not record the detailed medical history, physical examination, and biomarker analysis, including the injury onset, neurological examination, and serum lactate level.

## 5. Conclusions

In summary, the SI and SIPA are useful for identifying the compensatory phase of shock in prehospital and hospital settings, especially in corresponding normal to low-normal blood pressure. SIPA is effective for predicting the mortality and severity of traumatic injuries in the pediatric population. However, SI and SIPA were not significant predictors of ICU admission and need for surgery analysis.

## Figures and Tables

**Table 1:** Age-adjusted shock index cutoff value.

Age	Heart Rate	Systolic Blood Pressure	Shock Index Cutoff Value
≤3 years	70–110	90–110	1.2
4–6 years	65–110	90–110	1.2
7–12 years	60–100	100–120	1.0
>12 years	55–90	100–135	0.9

**Table 2:** Demographic population of all pediatric trauma patients.

Characteristics	All Pediatric Population
	N = 1265
Age (years), mean ± SD	14.27 ± 5.51
Male, n (%)	912(72.1%)
Underlying diseases, n (%)	70(5.5%)
Vital sign	
SBP, mean ± SD	126.73 ± 23.48
DBP, mean ± SD	75.33 ± 13.67
RR, mean ± SD	19.17 ± 2.58
HR, mean ± SD	93.56 ± 19.57
Triage	
I	96(7.6%)
II	698(55.2%)
III	467(36.9%)
IV + V	4(0.3%)
Consciousness level	
15	1152(91.1%)
8–15	88(7.0%)
≤8	24(1.9%)
Injury site	
Home	160(12.6%)
Street	760(60.1%)
Public site	268(21.2%)
Others	77(6.1%)
Mechanism	
Motor Vehicle Collision	672(53.1%)
Fall	387(30.6%)
Crushing injury	76(6.0%)
Sharp object	55(4.3%)
Others	75(5.9%)
Injured area	
Head and neck	251(18.8%)
Face	199(15.7%)
Thorax	82(6.5%)
Abdomen	91(7.2%)
Extremity	920(72.7%)
Activation of trauma team	58(4.6%)
Trauma scores	
Shock index	0.77 ± 0.24
Shock index ≥ 0.9	305(24.1%)
SIPA	0.86 ± 0.34
Elevated SIPA	171(13.5%)
ISS, (mean; SD)	6.51 ± 5.80
ISS ≥ 16, (%)	78(6.2%)
RTS, (mean; SD)	7.70 ± 0.63
NISS, (mean; SD)	7.36 ± 6.89
TRISS, (mean; SD)	0.98 ± 0.10
Clinical outcome	
LOS days, (median; IQR)	5.0 (3.0–9.0)
ICU Admission, (%)	177(14.0%)
ICU Readmission, (%)	2(0.2%)
ICU days, (median; IQR)	4.0 (2.0–6.0)
Need for surgery (%)	867(68.5%)
Reoperation (%)	49(3.9%)
Death, (%)	7(0.6%)

SD: standard deviation; IQR: interquartile range; SBP: Systolic blood pressure; DBP: diastolic blood pressure; RR: respiration rate; HR: heart rate; ISS: injury severity score; RTS: revised trauma score; NISS: National Industrial Security System; TRISS: new trauma and injury severity score; LOS days: length of stay days; and ICU: intensive care unit.

**Table 3:** Demographic population of elevated shock index and age-adjusted pediatric shock index.

Characteristics	SI < 0.9	SI ≥ 0.9	*p*-Value	Normal SIPA	Elevated SIPA	*p*-Value
	N = 960	N = 305		N = 1094	N = 171	
Age (years), mean ± SD	15.91 ± 4.04	9.09 ± 6.27	<0.001	14.63 ± 5.22	11.97 ± 6.69	<0.001
Male, n (%)	727(75.73%)	185(60.66%)	<0.001	808(73.86%)	104(60.82%)	<0.001
Underlying diseases, n (%)	58(6.04%)	12(3.93%)	0.161	63(5.76%)	7(4.09%)	0.376
Vital sign						
SBP, mean ± SD	133.69 ± 20.63	104.83 ± 17.78	<0.001	130.67 ± 21.64	101.53 ± 18.74	<0.001
DBP, mean ± SD	78.07 ± 12.24	66.70 ± 14.33	<0.001	77.01 ± 12.87	64.54 ± 13.69	<0.001
RR, mean ± SD	18.78 ± 2.0	20.39 ± 3.61	<0.001	19.01 ± 2.23	20.17 ± 4.06	<0.001
HR, mean ± SD	87.19 ± 14.53	113.62 ± 19.85	<0.001	90.05 ± 16.83	116.06 ± 20.90	<0.001
Injury site						
Home	72(7.50%)	88(28.85%)	<0.001	125(11.43%)	35(20.47%)	<0.001
Street	631(65.73%)	129(42.30%)	<0.001	666(60.88%)	94(54.97%)	0.143
Public site	86(8.96%)	29(9.51%)	0.771	104(9.51%)	11(6.43%)	0.194
Others	165(17.19%)	56(18.36%)	0.638	191(17.46%)	30(17.54%)	0.978
ISS, (mean; SD)	6.33 ± 5.28	7.10 ± 7.20	0.084	6.22 ± 5.08	8.39 ± 8.98	0.002
ISS ≥ 16, (%)	46(4.79%)	32(10.49%)	<0.001	52(4.75%)	26(15.20%)	<0.001
RTS, (mean; SD)	7.75 ± 0.52	7.53 ± 0.87	<0.001	7.74 ± 0.55	7.42 ± 0.94	<0.001
NISS, (mean; SD)	7.17 ± 6.32	7.97 ± 8.42	0.126	7.04 ± 6.08	9.40 ± 10.49	0.005
TRISS, (mean; SD)	0.98 ± 0.09	0.97 ± 0.13	0.091	0.98 ± 0.09	0.97 ± 0.12	0.152
LOS days, (median; IQR)	5.0 (3.0–9.0)	3.0 (2.0–7.0)	<0.001	5.0(3.0–9.0)	4.0(2.0–10.0)	0.204
ICU admission, (%)	137(14.27%)	40(13.11%)	0.612	162(14.81%)	15(8.77%)	0.034
ICU readmission, (%)	2(0.21%)	0(0.00%)	1.000	2(0.18%)	0(0.00%)	1.000
ICU days, (median; IQR)	4.0 (3.0–6.0)	3.5 (2.0–6.0)	0.556	4.0(2.0–6.0)	3.0(2.0–5.0)	0.411
Need for surgery (%)	664(69.17%)	203(66.56%)	0.363	753(68.83%)	114(66.67%)	0.571
Reoperation (%)	35(3.65%)	14(4.59%)	0.457	44(4.02%)	5(2.92%)	0.489
Death, (%)	4(0.42%)	3(0.98%)	0.369	4(0.37%)	3(1.75%)	0.056

SD: standard deviation; IQR: interquartile range; SBP: Systolic blood pressure; DBP: diastolic blood pressure; RR: respiration rate; HR: heart rate; ISS: injury severity score; RTS: revised trauma score; NISS: National Industrial Security System; TRISS: new trauma and injury severity score; LOS days: length of stay days; and ICU: intensive care unit.

**Table 4:** Unadjusted odds ratio for mortality related to in-hospital parameters.

Characteristics	Crude OR of Mortality			Crude OR of ICU Admission			Crude OR of Need for Surgery		
	OR	95% CI	*p*-Value	OR	95% CI	*p*-Value	OR	95% CI	*p*-Value
Age	1.245	0.943–1.644	0.121	1.000	0.971–1.029	0.987	1.022	1.001–1.044	0.040
Male	0.967	0.187–5.008	0.968	1.082	0.756–1.550	0.666	0.928	0.711–1.211	0.584
Underlying diseases, n (%)	13.332	2.925–60.777	<0.001	1.156	0.595–2.246	0.669	0.765	0.464–1.261	0.294
Vital sign									
SBP	0.985	0.953–1.018	0.355	0.999	0.992–1.006	0.716	1.003	0.998–1.009	0.190
DBP	0.937	0.885–0.991	0.023	0.992	0.980–1.003	0.153	1.002	0.993–1.010	0.732
RR	0.850	0.617–1.170	0.319	1.026	0.968–1.088	0.384	1.023	0.976–1.073	0.338
HR	1.006	0.971–1.043	0.726	0.998	0.989–1.006	0.565	1.003	0.997–1.009	0.309
Shock index	7.991	1.033–61.838	0.047	0.853	0.434–1.674	0.644	0.945	0.577–1.545	0.820
Shock index > 0.9	2.375	0.529–10.668	0.259	0.907	0.621–1.324	0.612	0.887	0.674–1.167	0.393
Elevated SIPA	4.867	1.080–21.937	0.039	0.553	0.317–0.964	0.038	0.906	0.643–1.276	0.571
ISS	1.135	1.084–1.189	<0.001	0.996	0.968–1.025	0.785	0.987	0.967–1.006	0.180
ISS ≥ 16	98.833	11.741–831.938	<0.001	0.791	0.388–1.615	0.520	0.719	0.448–1.153	0.171
RTS	0.521	0.395–0.689	<0.001	1.028	0.788–1.341	0.840	1.173	0.980–1.405	0.083
NISS	1.107	1.069–1.145	<0.001	1.002	0.979–1.024	0.896	0.993	0.977–1.010	0.425
TRISS	0.016	0.002–0.106	<0.001	3.741	0.303–46.183	0.304	1.173	0.360–3.823	0.791
Activation of trauma team	56.839	10.778–299.750	<0.001	1.136	0.548–2.355	0.732	0.548	0.322–0.933	0.027
LOS days	1.004	0.972–1.037	0.803	1.035	1.021–1.049	<0.001	0.995	0.986–1.004	0.272

SBP: Systolic blood pressure; DBP: diastolic blood pressure; RR: respiration rate; HR: heart rate; ISS: injury severity score; RTS: revised trauma score; NISS: National Industrial Security System; TRISS: new trauma and injury severity score; and SIPA: pediatric age-adjusted shock index.

**Table 5:** Subgroup analysis of elevated shock index and age-adjusted pediatric shock index in different age ranges.

Characteristics	Age ≤ 8 year	8 ≤ Age < 12 year	12 ≤ Age < 16 year	16 ≤ Age < 20 year	*p*-Value
	N = 236	N = 165	N = 237	N = 627	
Age (years), mean ± SD	4.69 ± 2.38	10.65 ± 1.11	14.74 ± 1.16	18.65 ± 1.03	
Male, n (%)	150(36.4%)	113(31.5%)	183(22.9%)	466(25.7%)	0.003
Underlying diseases, n (%)	9(3.8%)	10(6.1%)	13(5.5%)	38(6.1%)	0.624
Vital sign					
SBP, mean ± SD	108.82 ± 19.87	120.5 ± 18.30	131.50 ± 21.56	133.29 ± 22.80	<0.001
DBP, mean ± SD	69.36 ± 15.90	74.52 ± 11.33	75.59 ± 12.35	77.69 ± 13.11	<0.001
RR, mean ± SD	20.78 ± 3.83	18.99 ± 2.05	18.73 ± 1.85	18.76 ± 2.08	<0.001
HR, mean ± SD	109.40 ± 21.23	93.45 ± 15.53	88.93 ± 16.31	89.38 ± 17.91	<0.001
Injury site					
Home	92(39.0%)	28(17.0%)	16(6.8%)	24(3.8%)	<0.001
Street	64(27.1%)	51(30.9%)	129(54.7%)	516(82.3%)	<0.001
Public site	26(11.0%)	25(15.2%)	41(17.4%)	23(3.7%)	<0.001
Others	51(21.6%)	61(37.0%)	50(20.8%)	59(9.4%)	<0.001
ISS, (mean; SD)	5.34 ± 3.40	4.90 ± 2.89	6.70 ± 6.71	7.31 ± 6.54	<0.001
ISS ≥ 16, (%)	12(5.1%)	5(3.0%)	16(6.8%)	45(7.2%)	0.210
RTS, (mean; SD)	7.59 ± 0.83	7.80 ± 0.20	7.70 ± 0.55	7.70 ± 0.64	<0.001
NISS, (mean; SD)	5.88 ± 3.98	5.67 ± 3.39	7.72 ± 8.05	8.24 ± 7.75	<0.001
TRISS, (mean; SD)	0.97 ± 0.15	1.00 ± 0.00	0.98 ± 0.10	0.98 ± 0.09	<0.001
Shock index	1.04 ± 0.27	0.79 ± 0.17	0.69 ± 0.17	0.69 ± 0.19	<0.001
Shock index > 0.9	167(70.8%)	44(26.7%)	29(12.3%)	65(10.4%)	<0.001
Elevated SIPA	53(22.5%)	24(14.5%)	29(12.3%)	65(10.4%)	<0.001
LOS days, (median; IQR)	3(2–5)	3(2–5)	5(3–8)	6(4–11)	<0.001
ICU admission, (%)	33(14.0%)	20(12.1%)	36(15.3%)	88(14.0%)	0.858
ICU readmission, (%)	0(0.0%)	0(0.0%)	0(0.0%)	2(0.3%)	0.564
ICU days, (median; IQR)	4(2–6)	4(3–5.75)	4(3–7)	3(2–6)	0.303
Need for surgery (%)	148(62.7%)	114(69.1%)	165(69.9%)	440(70.2%)	0.200
Reoperation (%)	13(5.5%)	6(3.6%)	10(4.2%)	20(3.2%)	0.461
Death, (%)	0(0.0%)	0(0.0%)	3(1.3%)	4(0.6%)	0.212

SD: standard deviation; IQR: interquartile range; SBP: systolic blood pressure; DBP: diastolic blood pressure; RR: respiration rate; HR: heart rate; ISS: injury severity score; RTS: revised trauma score; NISS: National Industrial Security System; TRISS: new trauma and injury severity score; LOS days: length of stay days; and ICU: intensive care unit.

## Data Availability

Not applicable.
